# Macroscopic and deterministic quantum feature generation via phase basis quantization in a cascaded interferometric system

**DOI:** 10.1038/s41598-021-98478-8

**Published:** 2021-09-24

**Authors:** Byoung S. Ham

**Affiliations:** School of Electrical Engineering and Computer Science, Gwangju Institute of Science and Technology, 123 Chumdangwagi-ro, Buk-gu, Gwangju, 61005 South Korea

**Keywords:** Quantum mechanics, Quantum optics

## Abstract

Quantum entanglement is the quintessence of quantum information science governed by quantum superposition mostly limited to a microscopic regime. For practical applications, however, macroscopic entanglement has an essential benefit for quantum sensing and metrology to beat its classical counterpart. Recently, a coherence approach for entanglement generation has been proposed and demonstrated in a coupled interferometric system using classical laser light, where the quantum feature of entanglement has been achieved via phase basis superposition between identical interferometric systems. Such a coherence method is based on the wave nature of a photon without violating quantum mechanics under the complementarity theory. Here, a method of phase basis quantization via phase basis superposition is presented for macroscopic entanglement in an interferometric system, which is corresponding to the energy quantization of a photon.

## Introduction

Quantum entanglement^1^ has been understood as a mysterious entity in quantum mechanics over the last century limited to a microscopic regime governed by quantum superposition between two or more particles. Quantum entanglement has been implemented for various potential applications of quantum information science such as quantum computation^2–5^, quantum cryptography^6–8^, and quantum sensing^9–12^. In quantum sensing and quantum metrology, higher-order entangled states gives a great benefit due to correlated photon (atom) number-proportional sensitivity and imaging resolution. Although the higher-order entangled states, i.e., a N00N state, has been demonstrated for the photonic de Broglie waves (PBWs)^13–15^, its potential applications for quantum sensing have still been limited by the lower N number^15^. The inevitable limitation for the N number is due to probabilistic post-measurement process for coincidence detections confined by Poisson statistics or $\chi^{\left( 2 \right)}$ nonlinear processes, where the N-photon detection probability in Poisson statistics is proportional to $e^{ - N}$. The higher-order entangled photon generation by $\chi^{\left( 2 \right)}$ is even worse.

Complementarity theory or wave-particle duality is the core concept of quantum mechanics resulting from Copenhagen interpretation for a single particle^16^. Thus, energy quantization of the particle nature of a photon is incompatible with phase quantization of the wave nature. The energy quantization-based interpretation of quantum mechanics results in a phase information-independent measurement technique in a coupled system for such as anticorrelation, the co-called Hong-Ou-Mandel (HOM) dip^17^, and Bell inequality violation^18^ for nonlocal correlation between the paired particles. In an interferometric system of a Young’s double-slit system or a Mach–Zehnder interferometer (MZI), a single photon-based interference fringe is explained by self-interference satisfying the wave nature of Copenhagen interpretation. Such a wave property of a single photon is related with self-interference of a single photon under the Born’s rule^19^, and a related quantum test tool has been suggested by Sorkin in 1970s^20^. As claimed by Dirac^21^ and Feynman^22^, a photon never interferes or interacts with others but itself^23^. The concept of self-interference has expanded into a multi-photon-multi-slit system, where bipartite correlation is a part of it^24^.

Recently, wave nature-based quantum mechanical interpretations have been presented to understand fundamental physics of deterministic quantum feature generation for such as anticorrelation (a HOM dip)^25^ and PBW in the name of coherence de Broglie waves (CBWs)^26^. For both cases, experimental demonstrations have also been followed by using coherent photons of an attenuated laser light^27^ and a typical laser itself without attenuation^28^, respectively. The wave nature approach for quantum features leads to an intrinsic property of determinacy in quantum feature generation, where phase-dependent anticorrelation has been analyzed for a typical spontaneous parametric down conversion-based HOM dip result^29^. According to the particle nature-based quantum interpretations, all measured HOM dips so far have no phase information between the paired photons. Regarding CBW, tensor product-based phase bases of bipartite photons in a coupled MZIs have been investigated for the origin of quantum feature, whose image resolution is enhanced by the number of MZIs. In CBWs, thus, the resulting phase bases can be interpreted as phase quantization, which corresponds to energy quantization of a N00N state in PBWs. Here, a fundamental physics of CBWs in an n-coupled MZIs is investigated for a general understanding as to what caused such a nonclassical feature and how to generate it. This understanding on CBWs paves a road to macroscopic quantum sensing based on phase quantization in a coupled interferometric system. In this case, the phase control of each MZI for CBWs is compatible with coincident photon number detection in PBWs.

## Results

**Figure 1:**
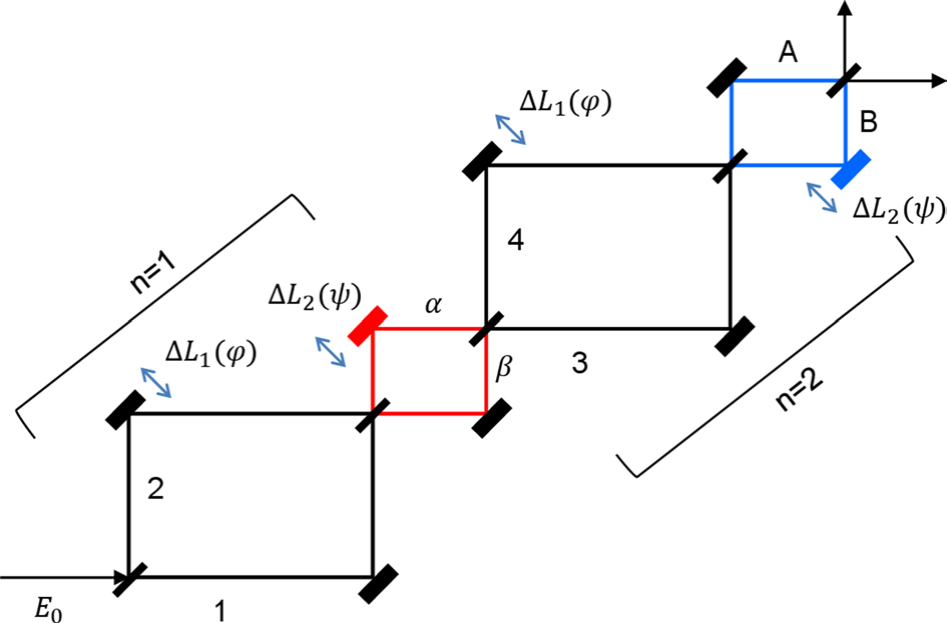
Schematic of macroscopic entanglement generation via path superposition of coupled interferometers. ${{\upvarphi }} = \frac{2\pi }{\lambda }{\Delta }L_{1}$; $\psi = \frac{2\pi }{\lambda }{\Delta }L_{2}$.

**Figure 2:**
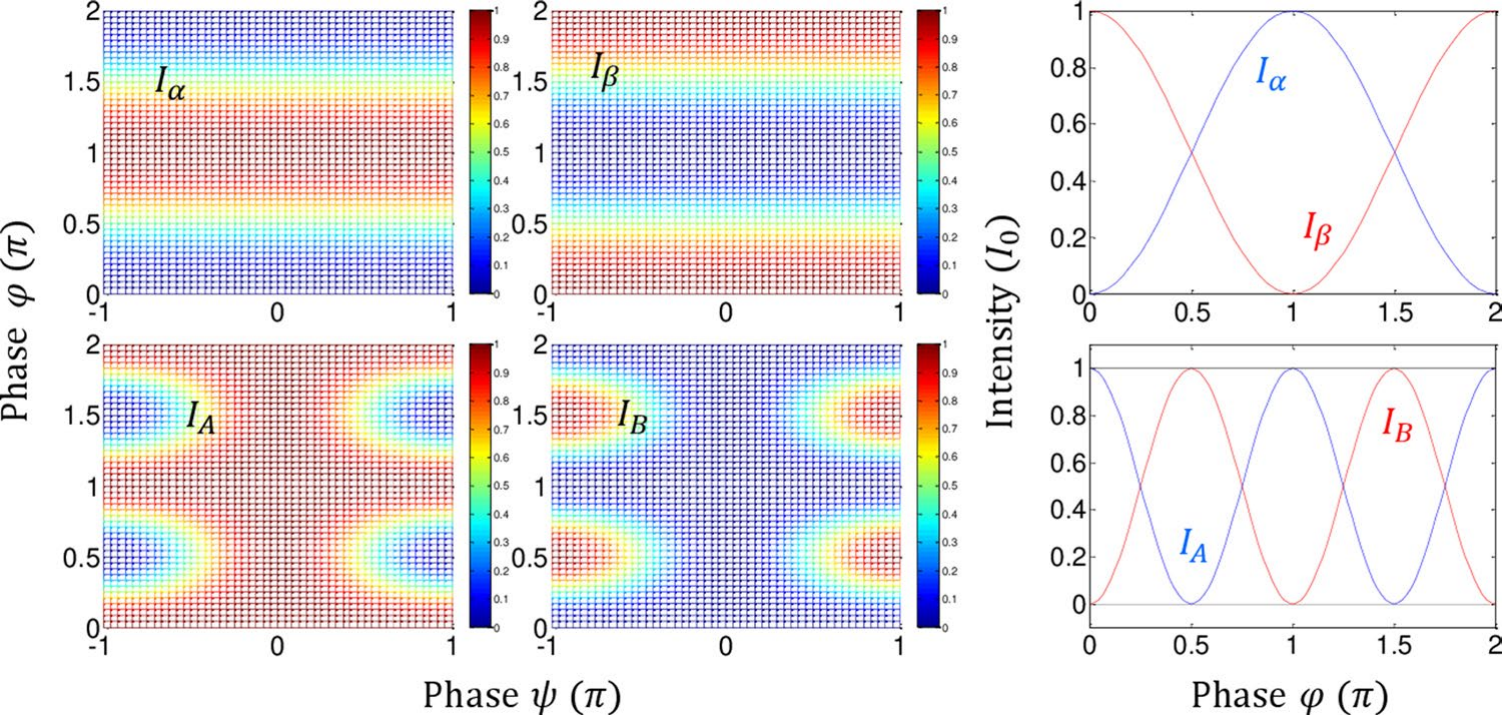
Numerical calculations for Fig. 1. Upper panels: n = 1. Lower panels: n = 2. (third column) Blue dotted and red curves: ${\uppsi } = \pm {\uppi }$. Black dotted (solid) line: *I*_*A*_ (*I*_*B*_) for ${\uppsi } = 0$.

Figure 1 shows a schematic diagram of an n-coupled MZIs for the macroscopic quantum feature of CBWs based on the on-demand phase control of ${{\upvarphi }}$ and $\psi$. For this, the basic building block is denoted by n = 1, where the paired MZIs having a common phase ${{\upvarphi }}$ are coupled via a dummy MZI with the phase ${\uppsi }$ (see the colored MZIs). In Fig. 1, both phase and intensity fluctuations of the coherent field *E*_0_ do not affect the final outputs, resulting in a robust quantum system. This is due to the BS matrix, where the split fields “1” and “2” in Fig. 1 are equal in measurements due to the Born’s rule of probability amplitudes^19^, where the BS always results in the fixed phase difference of $\frac{{\uppi }}{2}$ and the same intensity ratio between the split fields^25^. According to MZI physics, the output fields of the first ${{\upvarphi }} -$ MZI via the dummy MZI (red) in Fig. 1 are given by:

1 $$\left[ {\begin{array}{*{20}c} {E_{\alpha } } \\ {E_{\beta } } \\ \end{array} } \right] = \frac{1}{2}\left[ {\begin{array}{*{20}c} {e^{i\psi } \left( {1 - e^{i\varphi } } \right)} & {ie^{i\psi } \left( {1 + e^{i\varphi } } \right)} \\ {i\left( {1 + e^{i\varphi } } \right)} & { - \left( {1 - e^{i\varphi } } \right)} \\ \end{array} } \right]\left[ {\begin{array}{*{20}c} {E_{0} } \\ 0 \\ \end{array} } \right],$$

where the outputs are deterministic according to the phase basis of ${{\upvarphi }} \in \left\{ {0,\pi } \right\}$. The input field is coherent light from a typical laser: ${\varvec{E}}_{0} \left( {r,t} \right) = \left| {{\varvec{E}}_{0} } \right|e^{{i\left( {{\varvec{k}}_{0} {\varvec{r}} - 2\pi f_{0} t} \right)}}$. From Eq. (1), the corresponding intensities represent a classical bound governed by the Rayleigh criterion or a diffraction limit, whose maximum image resolution is ${\uplambda }/2$, in which the ${\uplambda }$ is the wavelength of the input field *E*_0_ (see the upper panels of Fig. 2):

2 $$I_{\alpha } = \frac{1}{2}\left( {1 - \cos \varphi } \right)I_{0} ,$$

3 $$I_{\beta } = \frac{1}{2}\left( {1 + \cos \varphi } \right)I_{0} ,$$

where $I_{0} = E_{0} E_{0}^{*}$. Unlike previous analysis with asymmetric coupling between ${{\upvarphi }} -$ MZIs with an opposite position of ${\Delta }L_{1}$ between n = 1 and n = 2 MZIs^26^, Fig. 1 is set for the same ${\Delta }L_{1}$ representing identically paired ${{\upvarphi }}$-MZIs, while an asymmetric ${\uppsi }$ is applied for the path superposition between them. In both cases, the asymmetry between neighboring MZIs is the essential condition of CBWs, where the ${\uppsi }$ positions results the identical output relation for ${\uppsi } = \pm {\uppi }$ due to ${{\cos}}\left( {\uppi } \right) = {{\cos}}\left( { - {\uppi }} \right)$ (see Fig. 2 and Sections A and B in the Supplementary Information). The diffraction limit as a classical bound in a typical MZI is clearly shown by the phase resolution of $\frac{\lambda }{2} \left( {{\text{or}}\;\pi } \right)$ in the upper right panel in Fig. 2.

For the doubly coupled MZIs via the asymmetric ${\uppsi }$ in Fig. 1, the output fields are represented as:

4 $$\left[ {\begin{array}{*{20}c} {E_{A} } \\ {E_{B} } \\ \end{array} } \right] = \frac{1}{4}\left[ {\begin{array}{*{20}c} {e^{i\psi } \left( {1 - e^{i\varphi } } \right)^{2} - \left( {1 + e^{i\varphi } } \right)^{2} } & {i\left( {e^{i\psi } - 1} \right)\left( {1 - e^{i2\varphi } } \right)} \\ {i\left( {e^{i\psi } - 1} \right)\left( {1 - e^{i2\varphi } } \right)} & { - e^{i\psi } \left( {1 - e^{i\varphi } } \right)^{2} + \left( {1 + e^{i\varphi } } \right)^{2} } \\ \end{array} } \right]\left[ {\begin{array}{*{20}c} {E_{0} } \\ 0 \\ \end{array} } \right],$$

where the corresponding output intensities of Eq. (4) are as follows (see the Supplementary Information):

5 $$I_{A} = \frac{1}{2}\left[ {1 + \cos \varphi^{2} + \sin \varphi^{2} \cos \psi } \right],$$

6 $$I_{B} = \frac{1}{4}\left[ {\left( {1 - \cos 2\varphi } \right)\left( {1 - \cos \psi } \right)} \right].$$

**Figure 3:**
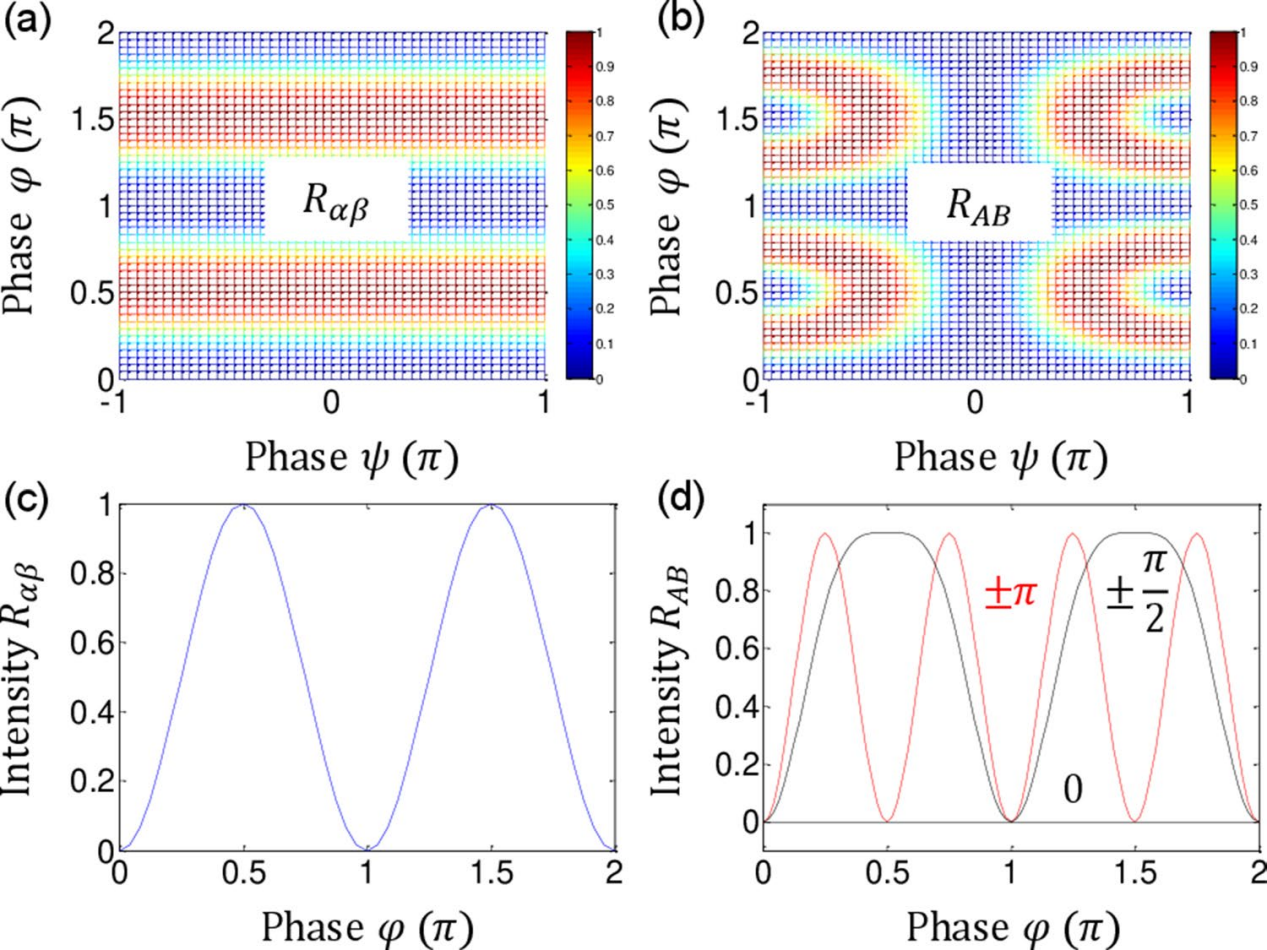
Numerical calculations for normalized intensity product *R*_*ij*_ for Fig. 2. (**a**) $R_{\alpha \beta }$. (**b**) $R_{\alpha \beta }$. (**c**) $R_{\alpha \beta }$ for ${\uppsi } = \pm {\uppi }$. (**d**) *R*_*AB*_ for ${\uppsi } = \pm {\pi }\left( {{\text{red}}} \right)$, ${\uppsi } = 0{ }\left( {{\text{dotted}}} \right)$, ${\uppsi } = \pm {\uppi }/2{ }\left( {{\text{black}}} \right)$.

From Eqs. (5) and (6), the following analyses are conducted for the ${\uppsi } -$ basis control (see the lower right panel in Fig. 2):

(i) For $${\uppsi } = 0$$,
  The output intensities of *I*_*A*_ and *I*_*B*_ are ${{\upvarphi }}$ independent, resulting in a fixed intensity as shown by the black dotted and solid lines for $I_{A} = I_{0}$ and $I_{B} = 0$, respectively (see the third column of Fig. 2). This identity relation results from double unitary transformations of CBWs for n = 2, and has been applied for a new type of quantum cryptography in the name of ‘unconditionally secured classical key distribution (USCKD)’^30^. Each ${{\upvarphi }}$-MZI plays a role of unitary transformation, where the dummy ${\uppsi }$-MZI reverses its time evolution of the wave function, resulting in identity relation in the final outputs through the followed ${{\upvarphi }}$-MZI (see Sect. 1 of the Supplementary Information of ref. ^30^). The USCKD can be explained as follows: The orthogonal phase basis choice of ${{\upvarphi }} \in \left\{ {0,\pi } \right\}$ assigned to Bob and Alice in the first and second ${{\upvarphi }}$-MZIs, respectively, in Fig. 1 results in the identity relation between the input and output fields for ${\uppsi } = 0$, as shown in Fig. 2. This is the deterministic randomness of USCKD, where the randomness results in the unconditional security via MZI superposition^30^.
(ii) For $$\psi = \pm \pi$$,
  The output intensities are ${{\upvarphi }}$ dependent, resulting in a swing property between the classical and quantum bounds: $I_{A} = I_{0} \left( {1 + \cos 2\varphi } \right)/2$ and $I_{B} = I_{0} \left( {1 - \cos 2\varphi } \right)/2$. In this case, the modulation term of $\cos 2\varphi$ cannot be obtained by classical physics limited by the Rayleigh criterion, which corresponds to a doubly increased frequency of *E*_0_. With a proper basis choice of ${\uppsi } \in \left\{ {0,\pi } \right\}$, thus, the phase resolution of output intensities is doubled due to the $\cos 2\varphi$ term as shown in the third column of Fig. 2. This MZI superposition-based nonclassical feature is called CBW^26,28^, where CBW is a wave version of quantum mechanics, corresponding to the particle version of PBW (discussed in Fig. 3)^14^. The interference fringe in the bottom row of Fig. 2 is completely different from multi-wave interference, e.g., Fabry–Perot, in classical physics. Thus, each fringe either *I*_*A*_ or *I*_*B*_ behaves such as frequency doubled laser light, i.e., $\lambda_{o} /2$ or $2f_{0}$. Unlike the particle nature-based PBWs, the wave nature-based CBWs result from the path superposition between identical ${{\upvarphi }}$-MZIs via the dummy ${\uppsi }$-MZI. The role of ${\uppsi }$-MZI for the nonclassical feature generation in Eqs. (5) and (6) is discussed below. Due to the wave nature, macroscopic entanglement of CBWs is also an inherent property, where the photon number does not affect the quantum feature. The direct proof of entanglement between bipartite entities has been done by either a HOM dip or Bell inequality measurements. The nonclassicality of CBW between “3” and “4” in Fig. 1 is demonstrated in Fig. 2 for ${\uppsi } = \pm {\uppi }$ (see Fig. 3).
(iii) For $$\psi = \pm \pi /2$$,

The output intensities are ${{\upvarphi }}$ dependent but do not reach the nonclassical bound, where the phase resolution fluctuates across the diffraction limit (details are analyzed in Fig. 3): $I_{A} = I_{0} \left( {1 + \cos \varphi^{2} } \right)/2$ and $I_{A} = I_{0} \left( {1 - \cos \varphi } \right)/4$. Neither a single MZI nor a coupled MZI reaches the quantum state without proper path superposition^31^.

**Figure 4:**
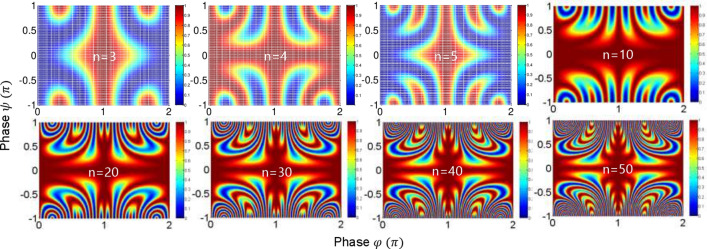
Numerical calculations for the output field intensity $I_{\alpha }^{\left( n \right)}$ for different n in Fig. 1.

**Figure 5:**
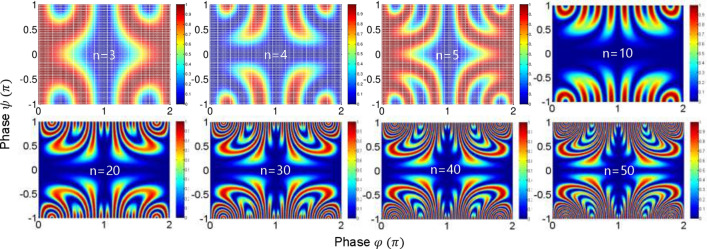
Numerical calculations for the output field intensity $I_{\beta }^{\left( n \right)}$ for different n in Fig. 1.

In a short summary, the $\cos 2\varphi$ modulation term in case (ii) for ${\uppsi } = \pm {\uppi }$ shows a definite quantum feature beating the classical limit of Rayleigh criterion in image resolution, where the MZI coupling method in Fig. 1 is via path (phase) superposition rather than conventional photon superposition in PBWs. Thus, the function of ${\uppsi }$-controlled superposition between two identical ${{\upvarphi }}$-MZIs decides whether the coupled system works for a classical one or a quantum one. The anticorrelation or entanglement condition is satisfied with a proper phase-basis choice^31,32^. To function as a quantum system, the phase-basis choice must be $\varphi_{AB} \in \left\{ {0,\frac{\pi }{2},\pi ,\frac{3\pi }{2}} \right\}$ for a coupled MZI as shown in the lower right corner of Fig. 2^32^. Here, the $\frac{\pi }{2}$ and $\frac{3\pi }{2}$ phase bases are newly created via double MZI coupling process in Fig. 1, where the phase basis quantization is $\varphi_{n} = \pi /n$ (discussed in Figs. 4 and 5).

Figure 2 shows numerical calculations for Fig. 1, where the upper panels are for a single MZI (n = 1) as a classical reference, while the lower panels are for a coupled ${{\upvarphi }}$-MZI via a dummy ${\uppsi }$-MZI (n = 2) for CBW. For the single MZI, the phase resolution is limited by the Rayleigh criterion at ${\uplambda }_{0} /2$. In the doubly coupled MZI, however, the phase resolution strongly depends on the choice of phase basis of ${\uppsi } \in \left\{ {0,\pi } \right\}$. If ${\uppsi } = 0$, the coupled system behaves as a perfect correlation system as shown by the dotted (solid) line in the lower right panel. If ${\uppsi } = \pm {\uppi }$, the coupled system swings between the quantum and classical bounds depending on ${{\upvarphi }}$-basis (discussed in Fig. 3).

Figure 3 shows normalized intensity product $R_{ij} \left( { = 4I_{i} I_{j} } \right)$ for Fig. 2. Figure 3a and b show the product *R*_*ij*_ as functions of ${{\upvarphi }}$ and ${\uppsi }$. Figure 3c and d are the details of Fig. 3a and b, respectively, where Fig. 3c represents the Rayleigh criterion as the conventional coherence limit in a single MZI comparable to N = 2 in the PBW^12,14^. The modulation frequency of *R*_*AB*_ in Fig. 3d varies between the classical and quantum bounds depending on ${\uppsi }$ values as analyzed above in Fig. 2. For ${\uppsi } = 0$ (see the dotted line in Fig. 3d), $R_{AB} = 0$ demonstrates an extreme bound of anticorrelation, representing a reversible process applied for USCKD^30^. In other words, the output direction in the doubly coupled MZI of Fig. 1 is predetermined depending on the ${\uppsi }$ basis via double unitary transformations. If ${\uppsi } = \pm {\uppi }$, *R*_*AB*_ swings between the quantum and classical bounds depending on the ${{\upvarphi }}$ values (see the red curve), resulting in a quantum feature with ${\uplambda }/8$ phase resolution, which is equivalent to the N = 4 in PBWs^14^. For the quantum sensing of CBWs, however, the output fields *I*_*A*_ and *I*_*B*_ do not need to be multiplied via an AND gate, where this AND gate operation of coincidence measurements is a necessary step for PBWs.

If ${\uppsi } = \pm {\uppi }/2$, it belongs somewhere between the classical and quantum bounds (see the green curve in Fig. 3d), partially violating the classical limit. Unlike the conventional understanding, coherence control by the dummy ${\uppsi }$-MZI for the coupled ${{\upvarphi }}$-MZIs in Fig. 1 functions as a decision maker for either a classical system or a quantum system. The major discovery in this manuscript is that entanglement is a deterministic feature for CBWs. Thus, the quantum feature of the present CBWs in a classically coupled MZI system can be manipulated deterministically and macroscopically via path superposition. This macroscopic and deterministic nonclassical property of CBWs do not violate the wave-particle duality in quantum mechanics^33^.

For generalized CBWs in a 2n-coupled ${{\upvarphi }} - {\uppsi }$ MZI system satisfying the CBW condition with ${\uppsi } =$ $\pm {\uppi }$, the following general output fields are obtained using Eq. (4) (see Sections A in the Supplementary Information):

7 $$\left[ {\begin{array}{*{20}c} {E_{\alpha } } \\ {E_{\beta } } \\ \end{array} } \right]^{\left( n \right)} = \left( { - 1} \right)^{n} \left( \frac{1}{2} \right)\left[ {\begin{array}{*{20}c} {\left( {1 + \left( { - 1} \right)^{n} e^{in\varphi } } \right)} & {i\left( {1 - \left( { - 1} \right)^{n} e^{in\varphi } } \right)} \\ { - i\left( {1 - \left( { - 1} \right)^{n} e^{in\varphi } } \right)} & {\left( {1 + \left( { - 1} \right)^{n} e^{in\varphi } } \right)} \\ \end{array} } \right]\left[ {\begin{array}{*{20}c} {E_{0} } \\ 0 \\ \end{array} } \right].$$

The respective output fields’ intensities of Eq. (7) are as follows (see Figs. 4 and 5):

8 $$I_{\alpha }^{\left( n \right)} = \frac{1}{2}I_{0} \left[ {1 + \left( { - 1} \right)^{n} \cos \left( {n\varphi } \right)} \right],$$

9 $$I_{\beta }^{\left( n \right)} = \frac{1}{2}I_{0} \left[ {1 - \left( { - 1} \right)^{n} \cos \left( {n\varphi } \right)} \right].$$

In Eqs. (8) and (9), the required condition for the quantum feature of anticorrelation^25^ is ${{\upvarphi }} = \pm \frac{m\pi }{n}$ (m = 0,1,2, …, n), as shown in Figs. 4 and 5. Under this condition, the phase resolution of each output field is $\delta \varphi_{n} = \lambda_{0} /2n$. Thus, quantum sensing is satisfied for all ${\text{n}} \ge 2$ to beat the classical limit of $\lambda_{0} /2$. As a result, the principal phase bases of the n-coupled MZIs are 0 and $\pi /n$, where $\pi /n$ represents the phase basis quantization. This is the phase quantization of CBWs corresponding to energy quantization in the particle nature of a photon for PBWs. This relation is bedrock to the enhanced phase resolution in the CBWs represented by $\lambda_{CBW} = \lambda_{0} /2{\text{n}}$ ^26,28,32^. As is well understood, the enhanced phase resolution results from the tensor product between n-bipartite MZIs^32^. A proper superposition for the tensor product plays a critical role as shown in Figs. 2, 3, 4 and 5.

The normalized intensity product $R_{\alpha \beta }^{\left( n \right)}$ is given by $\langle I_{\alpha }^{\left( n \right)} I_{\beta }^{\left( n \right)}\rangle /\langle I_{0}\rangle^{2}$:

10 $$R_{\alpha \beta }^{\left( n \right)} = \frac{1}{2}\langle 1 - \cos 2n\varphi \rangle.$$

Unlike the particle nature of photons heavily depending on measurement trials^9–15^, the average value in Eq. (10) is for a single shot measurement owing to the collective feature. For single shot measurements, multipartite entanglement generation has also been performed in a collective atomic ensemble^35,36^. For the CBW measurements, phase stabilization of the MZI system is a prerequisite, where such MZI stabilization has already been satisfied even for a km range of MZI system^34^. Thus, the phase resolution in the n-coupled MZI system of Fig. 1 is enhanced by a factor of n, where n = 1 is for the Rayleigh criterion or diffraction limit in classical physics (see Fig. 3c). Considering that the intensity correlation of coherent lights is $g^{\left( 2 \right)} \left( 0 \right) = 1$ due to Poisson statistics, the normalized intensity product in Eq. (10) violates the Poisson statistics-based classical result if $\varphi = \pm \frac{m\pi }{n}$ under the coupling condition of ${\uppsi } = \pm {\uppi }$. A detailed method for intensity correlation $g^{\left( 2 \right)} < 0.5$ satisfying quantum features has been discussed in refs. ^29^ and ^31^, where application of randomness results in uniform output fields in average values of each final port, but distinctive fringe of anticorrelation $\left( {g^{\left( 2 \right)} < 0.5} \right)$ in their products.

## Conclusion

In conclusion, quantum features of CBWs were analyzed in a coupled interferometric scheme of MZIs via path superposition control, where the asymmetric coupling method via a dummy MZI plays an essential role. Most of all, the generated quantum features were deterministically and macroscopically controllable for on-demand quantum feature generations. In addition, such deterministic and macroscopic entanglement generation technique was linearly scalable for higher-order quantum features corresponding to N00N states. Unlike the general understanding of entangled photon pair-based nonclassical features such as a HOM dip and Bell inequality violation, coherent light could generate the same quantum features of CBWs via path superposition among asymmetrical coupled identical MZIs. These quantum features of CBWs beating classical limit of Rayleigh criterion were originated in the phase basis quantization resulting from coupled MZIs, where the asymmetric coupling plays a key role. The enhanced phase resolution for ${\text{n}} \ge 2$ is a direct evidence of the quantum feature for the CBWs based on the wave nature of photons in a n-coupled MZIs. For potential applications of CBWs, a quantum sensor with coherent light can be implemented in a phase controlled Si-based waveguide structure.

## Methods

The numerical calculations in Figs. 2, 3, 4 and 5 were performed by Matlab using the equations in the text. The data that support the findings of this study are available from the corresponding author upon reasonable request.

## Supplementary Information

Supplementary Information.
