# Supplementary material for: Macroscopic and deterministic quantum feature generation via phase basis quantization in a cascaded interferometric system
Source: Sci Rep. 2021 Sep 24;11:19058. doi: 10.1038/s41598-021-98478-8 (PMC8463619; doi:10.1038/s41598-021-98478-8)
Supplement: Supplementary file 1 — Supplementary Information. [file 41598_2021_98478_MOESM1_ESM.pdf]

Supplementary Information for

Macroscopic and deterministic quantum feature generation via phase basis quantization in a cascaded interferometric system by

Byoung S. Ham
GIST, S. Korea

Section A

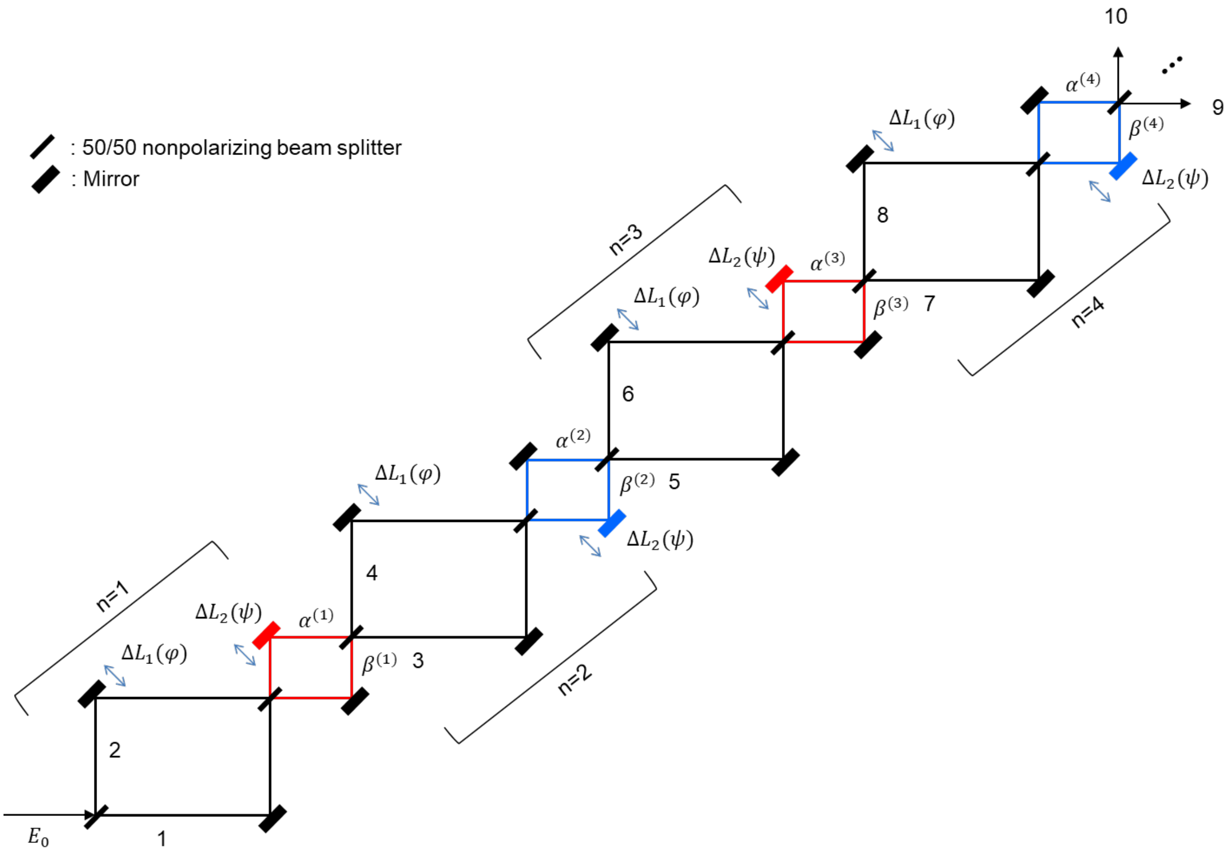

Fig. S1. Schematic of n-coupled CBWs.

(i) For n=1,

$$\begin{bmatrix}E_\alpha\\E_\beta\end{bmatrix} = \frac{1}{2}\begin{bmatrix}e^{i\psi}&0\\0&1\end{bmatrix}\begin{bmatrix}1&i\\i&1\end{bmatrix}\begin{bmatrix}1&0\\0&e^{i\varphi}\end{bmatrix}\begin{bmatrix}1&i\\i&1\end{bmatrix}\begin{bmatrix}E_0\\0\end{bmatrix} = \frac{1}{2}\begin{bmatrix}e^{i\psi}(1-e^{i\varphi}) & ie^{i\psi}(1+e^{i\varphi})\\ i(1+e^{i\varphi}) & -(1-e^{i\varphi})\end{bmatrix}\begin{bmatrix}E_0\\0\end{bmatrix}. \quad \text{(S1)}$$

(ii) For n=2,

$$\begin{bmatrix}E_\alpha\\E_\beta\end{bmatrix}^{(2)} = \begin{bmatrix}E_A\\E_B\end{bmatrix} = \frac{1}{2}\begin{bmatrix}1&0\\0&e^{i\psi}\end{bmatrix}\begin{bmatrix}1&i\\i&1\end{bmatrix}\begin{bmatrix}1&0\\0&e^{i\varphi}\end{bmatrix}\begin{bmatrix}1&i\\i&1\end{bmatrix}\begin{bmatrix}E_\alpha\\E_\beta\end{bmatrix}$$

$$= \frac{1}{4}\begin{bmatrix}(1-e^{i\varphi}) & i(1+e^{i\varphi})\\ ie^{i\psi}(1+e^{i\varphi}) & -e^{i\psi}(1-e^{i\varphi})\end{bmatrix}\begin{bmatrix}e^{i\psi}(1-e^{i\varphi}) & ie^{i\psi}(1+e^{i\varphi})\\ i(1+e^{i\varphi}) & -(1-e^{i\varphi})\end{bmatrix}\begin{bmatrix}E_0\\0\end{bmatrix}$$

$$= \frac{1}{4}\begin{bmatrix}e^{i\psi}(1-e^{i\varphi})^2-(1+e^{i\varphi})^2 & i\left[e^{i\psi}(1-e^{i2\varphi})-(1-e^{i2\varphi})\right]\\ ie^{i\psi}\left[e^{i\psi}(1-e^{i2\varphi})-(1-e^{i2\varphi})\right] & -e^{i\psi}\left[e^{i\psi}(1+e^{i\varphi})^2-(1-e^{i\varphi})^2\right]\end{bmatrix}\begin{bmatrix}E_0\\0\end{bmatrix}$$

$$= \frac{1}{4}\begin{bmatrix}e^{i\psi}(1-e^{i\varphi})^2-(1+e^{i\varphi})^2 & i\left[e^{i\psi}(1-e^{i2\varphi})-(1-e^{i2\varphi})\right]\\ ie^{i\psi}\left(e^{i\psi}-1\right)(1-e^{i2\varphi}) & -e^{i\psi}\left[e^{i\psi}(1+e^{i\varphi})^2-(1-e^{i\varphi})^2\right]\end{bmatrix}\begin{bmatrix}E_0\\0\end{bmatrix}. \quad \text{(S2)}$$

$$E_A = \frac{1}{4}\left[e^{i\psi}(1-e^{i\varphi})^2-(1+e^{i\varphi})^2\right], \quad \text{(S3)}$$

$$E_B = \frac{i}{4}e^{i\psi}\left[\left(e^{i\psi}-1\right)(1-e^{i2\varphi})\right], \quad \text{(S4)}$$

$$I_A = \frac{1}{16}\left[e^{i\psi}\left(1-e^{i\varphi}\right)\left(1-e^{i\varphi}\right)-\left(1+e^{i\varphi}\right)\left(1+e^{i\varphi}\right)\right]\left[e^{-i\psi}\left(1-e^{-i\varphi}\right)\left(1-e^{-i\varphi}\right)-\left(1+e^{-i\varphi}\right)\left(1+e^{-i\varphi}\right)\right]$$

$$= \frac{1}{16}\left[\left(1-e^{i\varphi}\right)\left(1-e^{-i\varphi}\right)\left(1-e^{i\varphi}\right)\left(1-e^{-i\varphi}\right)+\left(1+e^{i\varphi}\right)\left(1+e^{-i\varphi}\right)\left(1+e^{i\varphi}\right)\left(1+e^{-i\varphi}\right)-\right.$$

$$\left. e^{i\psi}\left(1-e^{i\varphi}\right)\left(1+e^{-i\varphi}\right)\left(1-e^{i\varphi}\right)\left(1+e^{-i\varphi}\right)-e^{-i\psi}\left(1+e^{i\varphi}\right)\left(1-e^{-i\varphi}\right)\left(1+e^{i\varphi}\right)\left(1-e^{-i\varphi}\right)\right]$$

$$= \frac{1}{16}\left[4(1-cos\varphi)^2+4(1+cos\varphi)^2-(-2isin\varphi)^2\left(e^{i\psi}+e^{-i\psi}\right)\right]$$

$$= \frac{1}{2}\left[1+(cos\varphi)^2+(sin\varphi)^2(cos\psi)\right]. \qquad \text{(S5)}$$

$$I_B = \frac{1}{16}\left[ie^{i\psi}\left(e^{i\psi}-1\right)\left(1-e^{i2\varphi}\right)\right]\left[-ie^{-i\psi}\left(e^{-i\psi}-1\right)\left(1-e^{-i2\varphi}\right)\right],$$

$$= \frac{1}{4}\left[(1-cos\psi)(1-cos2\varphi)\right]. \qquad \text{(S6)}$$

(iii) For n=3 with $\psi = \pm\pi$,

$$\begin{bmatrix} E_\alpha \\ E_\beta \end{bmatrix}^{(3)} = \left(\frac{1}{2}\right)^3 [M][M'][M] \begin{bmatrix} E_0 \\ 0 \end{bmatrix}$$

$$= \left(\frac{1}{2}\right)\begin{bmatrix} (1-e^{i3\varphi}) & i(1+e^{i3\varphi}) \\ -i(1+e^{i3\varphi}) & (1-e^{i3\varphi}) \end{bmatrix}\begin{bmatrix} E_0 \\ 0 \end{bmatrix}$$

where $[M] = \begin{bmatrix} -(1-e^{i\varphi}) & -i(1+e^{i\varphi}) \\ i(1+e^{i\varphi}) & -(1-e^{i\varphi}) \end{bmatrix}$ and $[M'] = \begin{bmatrix} (1-e^{i\varphi}) & i(1+e^{i\varphi}) \\ -i(1+e^{i\varphi}) & (1-e^{i\varphi}) \end{bmatrix}$. (S7)

(iv) For n=4 with $\psi = \pm\pi$,

$$\begin{bmatrix} E_\alpha \\ E_\beta \end{bmatrix}^{(4)} = \left(\frac{1}{2}\right)^4 [M'']^2 \begin{bmatrix} E_0 \\ 0 \end{bmatrix}$$

$$= \left(\frac{1}{2}\right)\begin{bmatrix} (1+e^{i4\varphi}) & i(1-e^{i4\varphi}) \\ -i(1-e^{i4\varphi}) & (1+e^{i4\varphi}) \end{bmatrix}^2\begin{bmatrix} E_0 \\ 0 \end{bmatrix}, \qquad \text{(S8)}$$

where $[M''] = [M'][M] = (-1)\left(\frac{1}{2}\right)\begin{bmatrix} (1+e^{i2\varphi}) & i(1-e^{i2\varphi}) \\ -i(1-e^{i2\varphi}) & (1+e^{i2\varphi}) \end{bmatrix}$.

(v) For n=5 with $\psi = \pm\pi$,

$$= (-1)\left(\frac{1}{2}\right)\begin{bmatrix} (1-e^{i5\varphi}) & i(1+e^{i5\varphi}) \\ -i(1+e^{i5\varphi}) & (1-e^{i5\varphi}) \end{bmatrix}. \qquad \text{(S9)}$$

Thus, the generalized solution for the n-coupled MZIs is as follows:

$$\begin{bmatrix} E_\alpha \\ E_\beta \end{bmatrix}^{(n)} = (-1)^n\left(\frac{1}{2}\right)\begin{bmatrix} (1+(-1)^n e^{in\varphi}) & i(1-(-1)^n e^{in\varphi}) \\ -i(1-(-1)^n e^{in\varphi}) & (1+(-1)^n e^{in\varphi}) \end{bmatrix}. \qquad \text{(S10)}$$

Section B

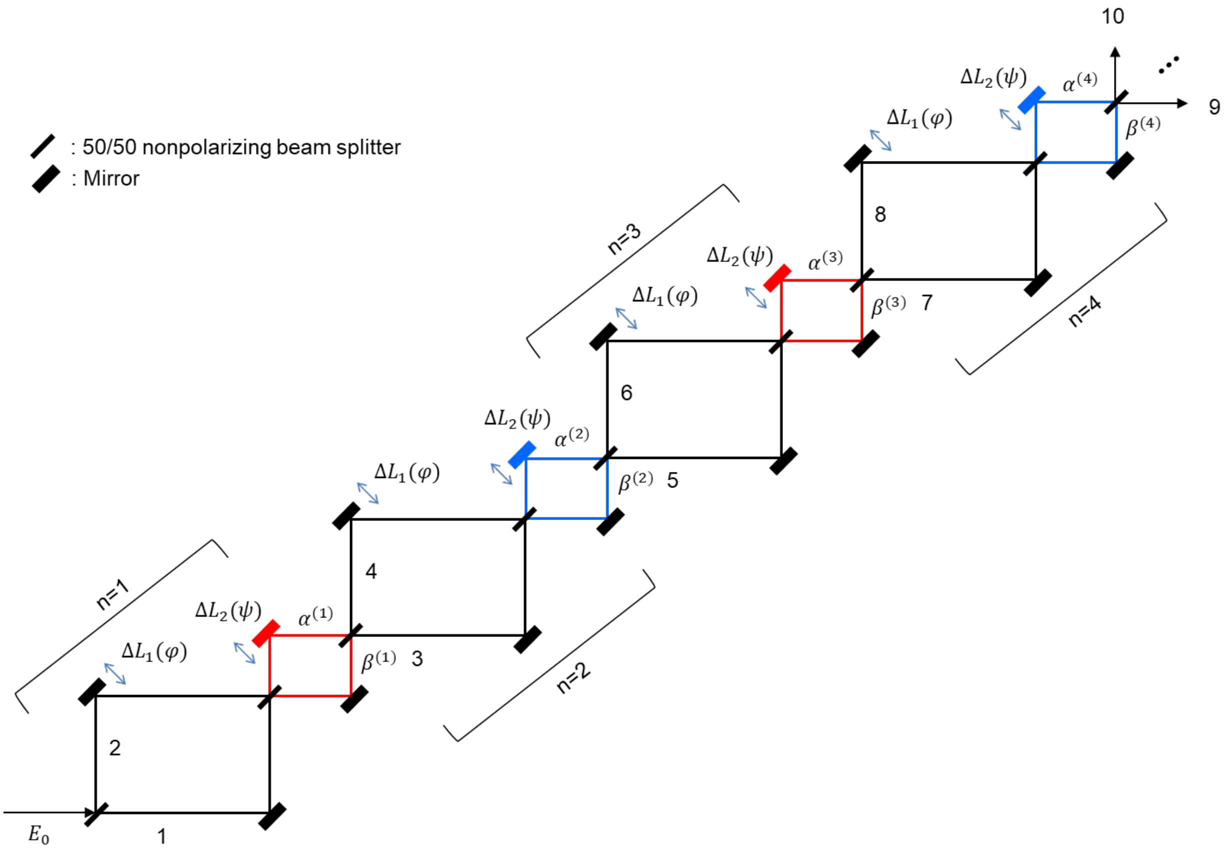

Fig. S2. Schematic of n-coupled CBWs.

(i) For n=1 with ψ = ±π,

$$\begin{bmatrix} E_\alpha \\ E_\beta \end{bmatrix} = \frac{1}{2}\begin{bmatrix} e^{i\psi} & 0 \\ 0 & 1 \end{bmatrix}\begin{bmatrix} 1 & i \\ i & 1 \end{bmatrix}\begin{bmatrix} 1 & 0 \\ 0 & e^{i\varphi} \end{bmatrix}\begin{bmatrix} 1 & i \\ i & 1 \end{bmatrix}\begin{bmatrix} E_0 \\ 0 \end{bmatrix}$$

$$= \frac{1}{2}\begin{bmatrix} e^{i\psi}(1-e^{i\varphi}) & ie^{i\psi}(1+e^{i\varphi}) \\ i(1+e^{i\varphi}) & -(1-e^{i\varphi}) \end{bmatrix}\begin{bmatrix} E_0 \\ 0 \end{bmatrix}$$

$$= \frac{1}{2}\begin{bmatrix} -(1-e^{i\varphi}) & -i(1+e^{i\varphi}) \\ i(1+e^{i\varphi}) & -(1-e^{i\varphi}) \end{bmatrix}\begin{bmatrix} E_0 \\ 0 \end{bmatrix}$$

$$= (-1)\frac{1}{2}\begin{bmatrix} (1-e^{i\varphi}) & i(1+e^{i\varphi}) \\ -i(1+e^{i\varphi}) & (1-e^{i\varphi}) \end{bmatrix}\begin{bmatrix} E_0 \\ 0 \end{bmatrix}. \qquad \text{(S11)}$$

Here, we define $[M] \equiv \begin{bmatrix} (1-e^{i\varphi}) & i(1+e^{i\varphi}) \\ -i(1+e^{i\varphi}) & (1-e^{i\varphi}) \end{bmatrix}$.

(ii) For n=2,

$$\begin{bmatrix} E_\alpha \\ E_\beta \end{bmatrix}^{(2)} = (-1)^2\left(\frac{1}{2}\right)^2([M])^2\begin{bmatrix} E_0 \\ 0 \end{bmatrix}$$

$$= \frac{1}{4}\begin{bmatrix} (1-e^{i\varphi}) & i(1+e^{i\varphi}) \\ -i(1+e^{i\varphi}) & (1-e^{i\varphi}) \end{bmatrix}\begin{bmatrix} (1-e^{i\varphi}) & i(1+e^{i\varphi}) \\ -i(1+e^{i\varphi}) & (1-e^{i\varphi}) \end{bmatrix}\begin{bmatrix} E_0 \\ 0 \end{bmatrix}$$

$$= \frac{1}{2}\begin{bmatrix} (1+e^{i2\varphi}) & i(1-e^{i2\varphi}) \\ -i(1-e^{i2\varphi}) & (1+e^{i2\varphi}) \end{bmatrix}\begin{bmatrix} E_0 \\ 0 \end{bmatrix}. \quad \text{(S12)}$$

(iii) For n=3 with $\psi = \pm\pi$,

$$\begin{bmatrix} E_\alpha \\ E_\beta \end{bmatrix}^{(3)} = (-1)^3\left(\frac{1}{2}\right)^3([M])^3\begin{bmatrix} E_0 \\ 0 \end{bmatrix}$$

$$= (-1)\left(\frac{1}{2}\right)\begin{bmatrix} (1-e^{i3\varphi}) & i(1+e^{i3\varphi}) \\ -i(1+e^{i3\varphi}) & (1-e^{i3\varphi}) \end{bmatrix}\begin{bmatrix} E_0 \\ 0 \end{bmatrix}. \quad \text{(S13)}$$

(iv) For n=4 with $\psi = \pm\pi$,

$$\begin{bmatrix} E_\alpha \\ E_\beta \end{bmatrix}^{(4)} = (-1)^4\left(\frac{1}{2}\right)^4([M])^4\begin{bmatrix} E_0 \\ 0 \end{bmatrix}$$

$$= \left(\frac{1}{2}\right)\begin{bmatrix} (1+e^{i4\varphi}) & i(1-e^{i4\varphi}) \\ -i(1-e^{i4\varphi}) & (1+e^{i4\varphi}) \end{bmatrix}^2\begin{bmatrix} E_0 \\ 0 \end{bmatrix}, \quad \text{(S14)}$$

(v) For n=5 with $\psi = \pm\pi$,

$$= (-1)\left(\frac{1}{2}\right)\begin{bmatrix} (1-e^{i5\varphi}) & i(1+e^{i5\varphi}) \\ -i(1+e^{i5\varphi}) & (1-e^{i5\varphi}) \end{bmatrix}. \quad \text{(S15)}$$

Thus, the generalized solution for the n-coupled MZIs is as follows:

$$\begin{bmatrix} E_\alpha \\ E_\beta \end{bmatrix}^{(n)} = (-1)^n\left(\frac{1}{2}\right)\begin{bmatrix} (1+(-1)^n e^{in\varphi}) & i(1-(-1)^n e^{in\varphi}) \\ -i(1-(-1)^n e^{in\varphi}) & (1+(-1)^n e^{in\varphi}) \end{bmatrix}. \quad \text{(S16)}$$
